# Progression of microstructural deterioration in load-bearing immobilization osteopenia

**DOI:** 10.1371/journal.pone.0275439

**Published:** 2022-11-04

**Authors:** Hironobu Koseki, Makoto Osaki, Yuichiro Honda, Shinya Sunagawa, Chieko Imai, Takayuki Shida, Umi Matsumura, Junya Sakamoto, Iku Tomonaga, Seiichi Yokoo, Satoshi Mizukami, Minoru Okita

**Affiliations:** 1 Department of Health Sciences, Nagasaki University Graduate School of Biomedical Sciences, Nagasaki, Japan; 2 Department of Orthopedic Surgery, Nagasaki University Graduate School of Biomedical Sciences, Nagasaki, Japan; 3 Department of Physical Therapy Science, Nagasaki University Graduate School of Biomedical Sciences, Nagasaki, Japan; 4 Department of Rehabilitation, Wajinkai Hospital, Nagasaki, Japan; 5 Department of Nursing, Fukuoka International University of Health and Welfare, Fukuoka, Japan; 6 Department of Public Health, Nagasaki University Graduate School of Biomedical Sciences, Nagasaki, Japan; University of Life Sciences in Lublin, POLAND

## Abstract

**Purpose:**

Immobilization osteopenia is a major healthcare problem in clinical and social medicine. However, the mechanisms underlying this bone pathology caused by immobilization under load-bearing conditions are not yet fully understood. This study aimed to evaluate sequential changes to the three-dimensional microstructure of bone in load-bearing immobilization osteopenia using a fixed-limb rat model.

**Materials and method:**

Eight-week-old specific-pathogen-free male Wistar rats were divided into an immobilized group and a control group (n = 60 each). Hind limbs in the immobilized group were fixed using orthopedic casts with fixation periods of 1, 2, 4, 8, and 12 weeks. Feeding and weight-bearing were freely permitted. Length of the right femur was measured after each fixation period and bone microstructure was analyzed by micro-computed tomography. The architectural parameters of cortical and cancellous bone were analyzed statistically.

**Results:**

Femoral length was significantly shorter in the immobilized group than in the control group after 2 weeks. Total area and marrow area were significantly lower in the immobilized group than in the control group from 1 to 12 weeks. Cortical bone area, cortical thickness, and polar moment of inertia decreased significantly after 2 weeks. Some cancellous bone parameters showed osteoporotic changes at 2 weeks after immobilization and the gap with the control group widened as the fixation period extended (*P* < 0.05).

**Conclusion:**

The present results indicate that load-bearing immobilization triggers early deterioration of microstructure in both cortical and cancellous bone after 2 weeks.

## Introduction

During fracture treatment, external fixation is applied to immobilize the bones and joints involved to stabilize and fix the fracture in both conservative and postoperative treatments. Periods of immobilization are well known to inevitably result in bone loss and the degradation of mechanical properties, both of which are associated with reduced bone strength and increased risk of fragility fractures [1, 2]. Bone normally undergoes a continual process of remodeling under the control of hormones and local cytokines, mediated by the coordinated actions of bone-resorbing osteoclasts and bone-forming osteoblasts. A prolonged lack of mechanical stress leads to osteopenia, in which bone-formation activities are not enhanced even in the presence of enhanced bone resorption [3–5]. Rather, bone formation is significantly suppressed in immobilization-induced osteopenia [6, 7]. Although this imbalance in remodeling is similar to the situation in osteoporosis, osteopenia may occur regardless of age, sex, estrogenic status, and steroid agents, resulting in bone loss and skeletal fragility [1, 2, 8]. Immobilization osteopenia therefore represents a critical pathological situation in which bone mass is continuously lost in the absence of any compensatory activity against bone reduction. While mechanical stress can be classified into stress from load-bearing and stress from muscle contractions, the effects of unloading on skeletal deterioration have been relatively well-discussed as “disuse osteopenia” for decades [1, 2]. Recently, interactions between muscle and bone (“crosstalk”) have gained attention as an important contributor to relevant bone loss, and both clinical studies of bone status for sarcopenia in the elderly [9] and basic research into the role of osteocytes have been undertaken [10]. However, the mechanisms and structural developments underlying immobilization-induced osteopenia solely due to a lack of muscle contractions remain unclear.

A guideline released by the United States Food and Drug Administration has appropriately underscored the need for rat experimentation regarding preclinical evaluation [11]. For rodent models of immobilization, studies have adopted nerve or spinal cord deficits, botulinum toxin, tail suspension and casting or bandaging of limbs [12, 13]. Such models elicit similar skeletal responses, with the predominant endpoint being site-specific bone loss. However, nerve or spinal deficits insulate centripetal sensory signals from tissues such as bone, joint, muscle, and skin, as well as efferent motor signals [14, 15]. Moreover, nerve resection might also induce phantom pain and autonomic dysfunction, which can affect bone metabolism. Nerve or spinal deficit models are therefore considered more suitable for localized immobilization due to paraplegia or paraparesis resulting from spinal cord injury, hemiplegia or hemiparesis due to stroke. Botulinum toxin inhibits transport of acetylcholine vesicles and temporarily prevents exocytosis of acetylcholine into the synaptic cleft [16]. In this model, gait ability is impaired and therefore both ground forces and muscle activity are abolished, similar to nerve or spinal cord deficit models. Tail suspension models eliminate the mechanical stress resulting from load-bearing, but maintain joint motion from muscle contractions. This model may thus be applicable to the rehabilitation of bedridden patients or training for astronauts under weightless or microgravity conditions [12, 13]. To investigate the effects of mechanical stress produced by muscle contractions on bone metabolism, cast immobilization is thought to be the most appropriate method [13]. Although some previous studies have adopted cast immobilization models, clear statements regarding load-bearing have been absent [17, 18]. Our casting model has been used for the assessment of muscle atrophy [19, 20], allowing evaluation of the effect of muscle contractions on bone. Temporal changes in the bone microstructure of immobilization osteopenia tolerating load-bearing have not previously been investigated.

The present study aimed to investigate sequential changes in the bone microstructure to determine bone quality and the architectural development processes in load-bearing immobilization osteopenia.

## Materials and methods

### Animals

One hundred and twenty 8-week-old, male specific-pathogen-free (SPF) Wistar rats (body weight, 220 ± 10 g at the start of the study) were purchased from CLEA Japan Inc. (Tokyo, Japan) and acclimated in a temperature- (25 ± 1°C), humidity- (50 ± 10%), and light-controlled (12 h light: 12 h dark cycle) animal housing facility. Animals were housed individually in sterilized cages for at least 1 week prior to the study for acclimatization to the environment, fed standard rodent chow containing 1.25% Ca and 1.06% phosphate (CE-2; CLEA Japan Inc.), and allowed free access to tap water.

### Experimental design

**Fig 1:**
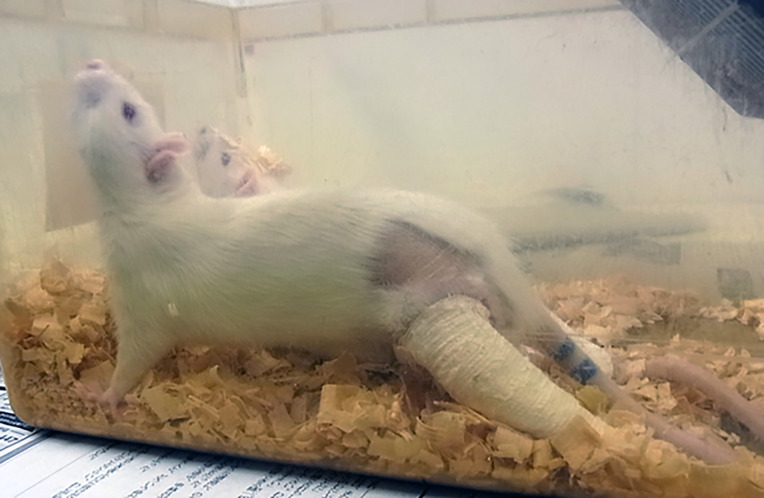
Immobilized animal model. Bilateral hind limbs of 8-week-old male SPF Wistar rats are fixed with the knee in a fully extended position using plaster casts.

This study adopted the previously described animal model of immobilization [19, 20]. We randomly divided the 120 rats into an immobilization group (Im group, n = 60) and a control group (n = 60). Rats were anesthetized by intraperitoneal injection with sodium pentobarbital (40 mg/kg), then bilateral hind limbs of rats in the Im group were fixed with the knee fully extended and the ankle in 40–45° of plantar flexion using plaster casts (Plasrun^®^; Alcare Co., Tokyo, Japan). The plaster cast was fitted from 1 cm below the hip joint to the distal foot and was changed every other day to prevent loosening with progression of muscle atrophy (Fig 1). Observations for skin abrasions and vein occlusions were performed at every cast rewinding. To exclude any effects of anesthesia, rats in the control group received anesthesia alone using the same timing. The immobilization period was set as 1, 2, 4, 8, or 12 weeks, with 12 rats used in each immobilization period. Immobilized rats could tolerate weight-bearing and could ambulate by moving the hip joint. Feeding and weight-bearing were freely permitted. After each fixation period, rats were sacrificed by an overdose of intraperitoneal sodium pentobarbital (200 mg/kg) under general anesthesia with 3% isoflurane (IsoFlo Vet; Orion Pharma Animal Health, Nivå, Denmark). After resecting the right femur, the soft tissue was removed and the bone was wrapped in gauze soaked in Ringer-Rock solution. The length of each femur was measured as the trochanter-malleolar distance using digital vernier calipers. The experimental protocol was approved by the Ethics Review Committee for Animal Experimentation at Nagasaki University Graduate School of Biomedical Sciences (approval no. 1409011170). All experimental procedures were performed under anesthesia in accordance with the Japan Government Principle for the Utilization and Care of Vertebrate Animals.

### Micro-CT imaging and 3-dimensional (3D) architectural indexes

Bone microstructure was evaluated using a high-resolution desktop micro-computed tomography (micro-CT) system (μCT40; Scanco Medical AG, Brüttisellen, Switzerland) in accordance with the American Society for Bone and Mineral Research guidelines for the use of micro-CT in rodents [21]. Individual femurs were stabilized in agarose in a scanning tube. After density calibration, scans were acquired using the following settings: isotropic voxel size, 12 μm^3^; peak X-ray tube potential, 70 kVp; peak X-ray tube intensity, 114 mA; and integration time, 200 ms. Scans were then subjected to Gaussian filtration. Cortical bone was evaluated in the mid-diaphysis in a region that started below the femoral head at 55% of bone length and extended 1.8 mm (150 slices) distally. Thresholds of 486 and 733 mg HA/cm^3^ were used for evaluating cortical bone, based on adaptive-iterative thresholding that was performed on the control group. Trabecular bone microarchitecture was evaluated in the distal metaphysis. The distal metaphyseal region analyzed began at 240 μm (20 slices) above the peak of the distal growth plate and extended 1.8 mm (150 slices) proximally. After micro-CT, original image data were transferred to a workstation, and structural indexes were calculated using 3D image analysis (TRI/3D-BON; Ratoc System Engineering, Tokyo, Japan). Cortical bone outcomes included total area (Tt.Ar, mm^2^), marrow area (Ma.Ar, mm^2^), cortical bone area (Ct.Ar, mm^2^), cortical bone area fraction (Ct.Ar/Tt.Ar, %), cortical thickness (Ct.Th, μm), and polar moment of inertia (pMOI, mm^4^). Cancellous bone outcomes included trabecular bone volume fraction (BV/TV, %), trabecular thickness (Tb.Th, μm), trabecular separation (Tb.Sp, μm), trabecular number (Tb.N, mm^-1^), structural model index (SMI), and degree of anisotropy (DA) as calculated using the distance transformation method [22]. SMI, defined as a value between 0 and 3, was used to estimate rod- and plate-like characteristics of trabecular structures. Plate-like trabeculae offer superior bone strength to rod-like trabeculae. DA, reflecting trabecular orientation, was determined from the ratio between maximal and minimal radii of the mean intercept length ellipsoid [23]. Connective density (Conn.D, mm^-3^) represents trabecular connectivity, directly indicating the state of trabecular connections [24].

### Statistical analysis

All data are expressed as median and interquartile range (IQR). Femoral length, and cortical and cancellous bone parameters in micro-CT were analyzed statistically. All variables were assessed for a normal distribution by the Shapiro-Wilk test. Comparisons between two groups were assessed using the Mann-Whitney U test. All data were analyzed using SPSS version 22.0 (SPSS, Chicago, IL, USA). Statistical significance was defined for values of *P* < 0.05.

## Results

### Femoral bone length

**Fig 2:**
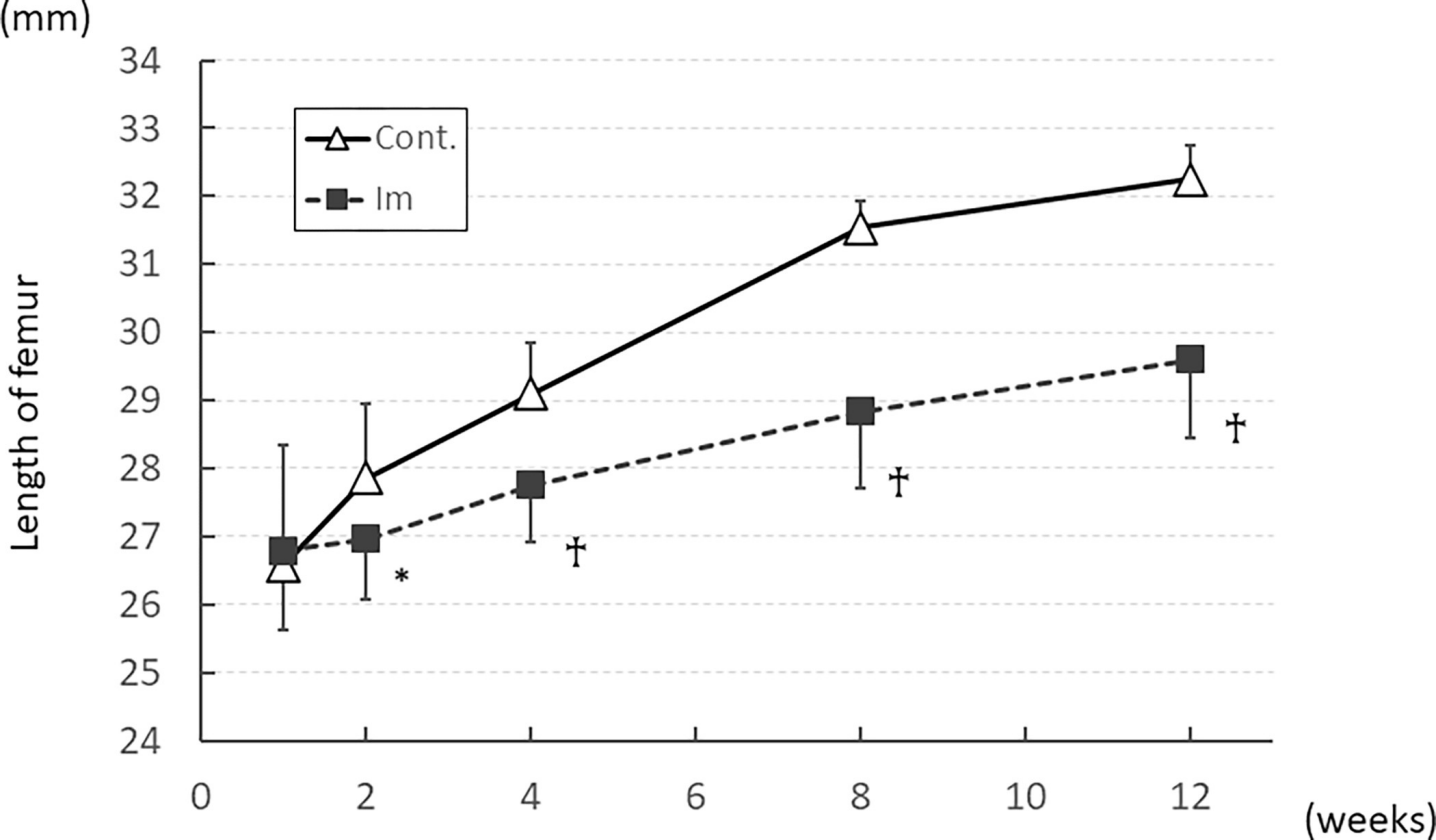
Temporal changes in length of the femur (trochanter-malleolar distance). Length of the femur was significantly shorter in the Im group than in the control group after 4 weeks, and this situation continued until the end of the experiment. ^†^ *P* < 0.01 compared to control group. * *P* < 0.05 compared to control group.

During the entire experiment, all rodent models survived until sacrifice. Although body weight in both groups increased with natural growth, rates in the Im group tended to be less than in the control group (S2 Data). Fig 2 shows the results for femur length. While bone length increased as experimental rodents matured, growth rates were significantly lower in the Im group than in the control group after 2 weeks of immobilization (*P* < 0.05).

### Microstructural parameters as determined by micro-CT

Parameters of cortical bone architecture defined by micro-CT are shown in Table 1. Values for Tt.Ar and Ma.Ar for the diaphysis became significantly lower in the Im group than in the control group from 1 week (-7.9% and -15.3%, respectively), and the significance of these differences persisted until the end of the study period (12 weeks). Although Ct.Ar was lower in the Im group than in the control group at the 2-week (-15.2%), 4-week (-20.3%), 8-week (-18.2%), and 12-week (-21.2%) time points, Ct.Ar/Tt.Ar did not change significantly throughout the experimental period. Ct.Th and pMOI were relatively less in the Im group after 2 weeks compared to control group (-10.5% and -22.7%, respectively) and these trends continued until 12 weeks.

**Table 1:** Cortical bone parameters in the midshaft of the femoral bone for the two groups at all time points.

		*1 week*	*2 weeks*	*4 weeks*	*8 weeks*	*12 weeks*
Tt.Ar (mm^2^)	Cont.	10.5 (10.2–11.0)	10.7 (10.2–11.0)	12.1 (11.8–13.1)	14.3 (13.0–15.0)	14.0 (13.7–14.3)
	Im	9.7 (9.4–10.1)*	9.7 (8.8–10.1)^♰^	9.7 (9.4–10.2)^♰^	12.0 (9.9–12.6)^♰^	11.8 (11.5–11.8)*
	vs. Cont.	92.1%	87.7%	78.0%	81.7%	85.2%
Ma.Ar (mm^2^)	Cont.	6.36 (5.49–7.81)	6.67 (5.81–6.95)	6.86 (6.74–7.45)	8.20 (7.81–8.76)	8.64 (8.50–8.77)
	Im	5.31 (5.20–5.95)*	5.24 (4.79–6.06)^♰^	5.71 (5.53–6.10)^♰^	6.98 (6.34–7.28)^♰^	6.83 (6.38–6.98)*
	vs. Cont.	84.7%	84.4%	80.0%	82.2%	79.1%
Ct.Ar (mm^2^)	Cont.	6.20 (5.32–6.34)	6.20 (5.99–6.34)	6.75 (6.61–7.33)	8.17 (7.71–8.64)	8.51 (8.38–8.65)
	Im	5.23 (5.07–5.86)	5.39 (5.00–5.56)^♰^	5.59 (5.41–5.97)^♰^	6.88 (6.21–7.18)^♰^	6.71 (6.25–6.87)*
	vs. Cont.	88.4%	84.8%	79.7%	81.8%	78.8%
Ct.Ar/Tt.Ar (%)	Cont.	57.4 (53.7–59.1)	54.0 (53.1–55.0)	56.0 (55.5–56.8)	57.6 (56.3–60.5)	62.6 (62.1–63.4)
	Im	53.8 (51.0–55.8)	52.6 (51.5–53.4)	56.2 (55.5–59.5)	58.3 (56.2–61.8)	54.6 (53.4–56.8)
	vs. Cont.	94.8%	97.2%	102.3%	100.3%	89.5%
Ct.Th (μm)	Cont.	533.0 (512.5–613.5)	590.8 (536.0–621.7)	634.0 (611.5–645.0)	726.0 (689.8–732.8)	781.0 (779.3–781.8)
	Im	506.5 (496.5–522.8)	525.5 (486.9–551.0)^♰^	564.5 (547.0–570.5)^♰^	648.0 (636.0–666.8)^♰^	637.0 (606.0–656.0)*
	vs. Cont.	89.8%	89.5%	89.9%	91.0%	82.4%
pMOI (mm^4^)	Cont.	14.7 (13.3–16.4)	14.5 (13.8–15.2)	19.0 (18.2–21.9)	26.2 (23.3–30.2)	25.9 (24.9–26.8)
	Im	12.5 (11.7–13.6)	11.7 (11.3–12.6)^♰^	12.5 (11.8–14.7)^♰^	19.0 (13.8–20.8)^♰^	17.5 (16.8–18.4)*
	vs. Cont.	87.0%	77.3%	62.0%	67.3%	72.7%

Data are presented as median (IQR) and percentage variation of the mean value with respect to control.

† *P* < 0.01 compared to control group

* *P* < 0.05 compared to control group

**Fig 3:**
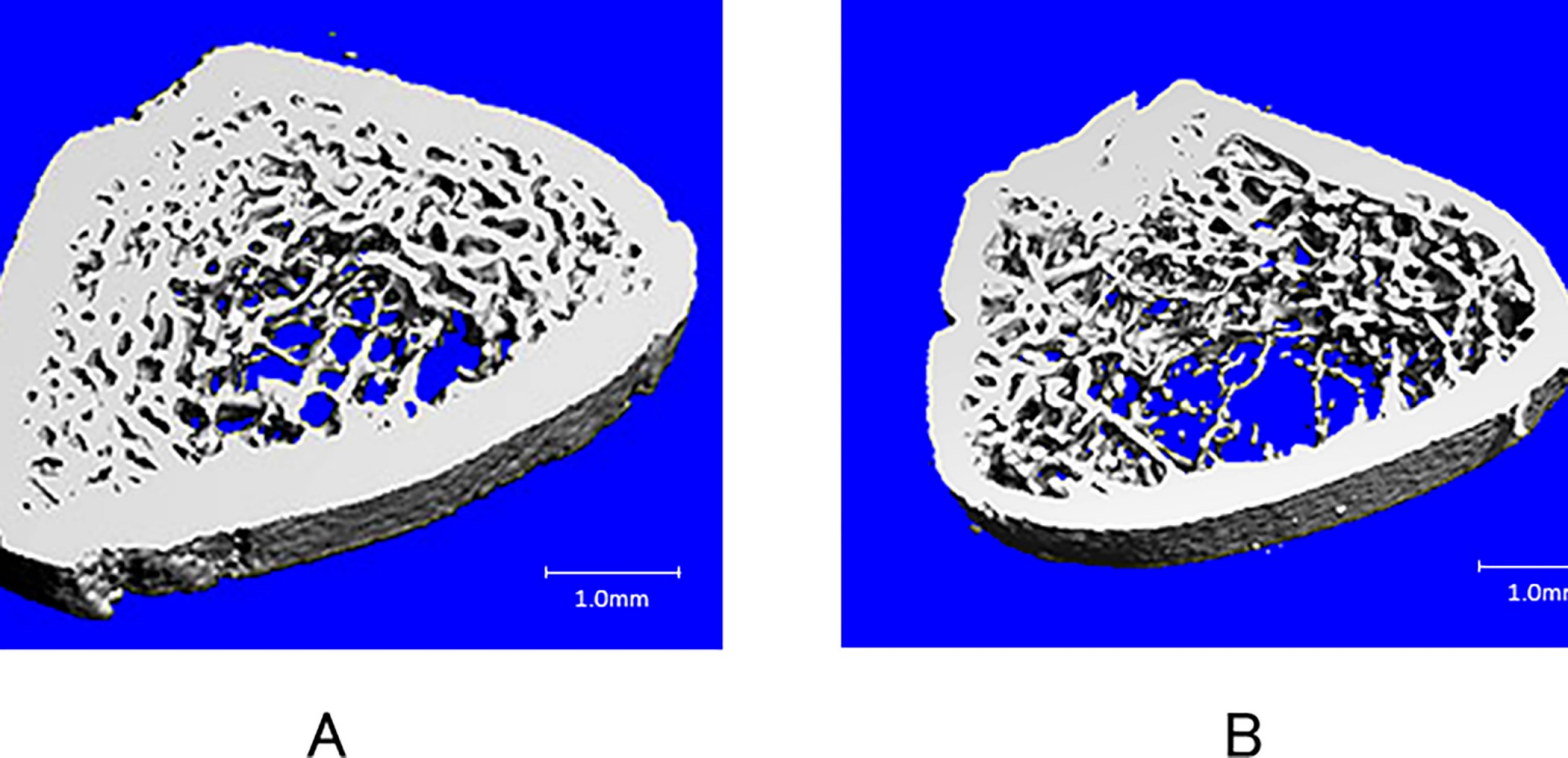
Typical 3D micro-CT images of the distal femoral metaphysis at week 12. Cortical bone was thin, and trabecular bone structures were smaller and fewer in the Im group compared to the control group. A: Control group; B: Im group. Scale bar = 1.0 mm.

Cancellous bone parameters showed that the Im group displayed loss of BV/TV and Tb.Th at 2 weeks (-28.4% and -8.8%, respectively), indicating the development of trabecular osteopenia in the femoral metaphysis. Tb.N followed a similar transition between groups until 8 weeks, but Tb.N was significantly lower in the Im group than in the control group at 12 weeks (-19.4%). SMI in the Im group hovered at a higher level compared to the control group, and significant differences were evident at 2, 4, 8, and 12 weeks, meaning that trabecular bone transformed from plate-like to rod-like structures. While DA in the Im group was inferior compared to the control group only at 8 weeks, DA in both groups gradually decreased similarly as the experimental period extended. Values of Tb.Sp and Conn.D in both groups shifted in the same way and no significant difference was evident between groups (Table 2). Visual inspection of registered images at 12 weeks showed thinning of cortical bone and sparse trabeculae, particularly in the core of bone marrow (Fig 3).

**Table 2:** Trabecular bone parameters in the distal femoral metaphysis for the two groups at all time points.

		*1 week*	*2 weeks*	*4 weeks*	*8 weeks*	*12 weeks*
BV/TV (%)	Cont.	34.8 (31.9–36.0)	39.9 (37.5–42.0)	35.4 (31.2–38.7)	37.3 (31.9–41.8)	47.9 (41.3–50.0)
	Im	31.8 (25.1–36.1)	31.5 (25.7–32.7)^♰^	26.6 (25.2–34.2)*	30.0 (27.2–34.3)*	28.5 (24.8–31.7)^♰^
	vs. Cont.	92.7%	71.6%	81.7%	81.8%	62.1%
Tb.Th (μm)	Cont.	94.6 (93.6–99.2)	106.4 (97.8–109.6)	101.5 (97.5–104.4)	111.6 (107.7–117.4)	126.1 (121.5–131.7)
	Im	93.6 (92.0–95.3)	98.8 (88.1–102.7)*	90.8 (88.1–95.6)^♰^	102.5 (94.1–107.2)^♰^	101.2 (96.2–103.2)^♰^
	vs. Cont.	97.0%	91.2%	90.2%	89.2%	79.5%
Tb.Sp (μm)	Cont.	201.4 (169.0–238.3)	178.8 (174.1–182.8)	217.7 (196.4–226.0)	247.7 (221.5–305.2)	218.3 (198.6–257.7)
	Im	225.3 (202.3–252.5)	182.2 (178.4–248.4)	217.3 (204.4–253.8)	245.2 (234.4–251.2)	274.1 (238.7–326.1)
	vs. Cont.	109.2%	114.9%	111.6%	96.3%	126.6%
Tb.N (mm^-1^)	Cont.	4.6 (4.0–5.1)	3.9 (3.6–4.7)	4.3 (4.1–4.6)	4.0 (3.4–4.3)	4.5 (3.9–4.8)
	Im	4.2 (3.8–4.5)	3.5 (3.4–3.7)	4.3 (3.7–4.5)	3.8 (3.7–4.0)	3.5 (3.0–4.0)*
	vs. Cont.	92.9%	86.8%	92.9%	97.9%	80.6%
SMI	Cont.	0.68 (0.47–1.00)	0.49 (0.35–0.90)	0.59 (0.23–1.22)	0.28 (0.06–0.68)	- 1.11 (-1.27–0.36)
	Im	1.02 (0.65–1.62)	1.69 (1.54–1.73)^♰^	1.50 (0.81–1.60)*	1.17 (0.88–1.48)^♰^	1.24 (0.90–1.64)^♰^
DA	Cont.	1.99 (1.95–2.02)	1.77 (1.71–2.13)	1.83 (1.80–2.00)	1.78 (1.75–1.80)	1.67 (1.63–1.75)
	Im	1.95 (1.71–2.11)	1.79 (1.73–1.93)	1.96 (1.77–2.01)	1.69 (1.65–1.74)^♰^	1.66 (1.64–1.69)
Conn.D (mm^-3^)	Cont.	81.6 (75.1–91.5)	93.4 (90.6–94.3)	78.6 (72.4–87.6)	73.3 (66.4–77.2)	76.8 (68.2–81.7)
	Im	88.9 (69.4–98.9)	87.9 (65.5–107.5)	75.6 (70.0–80.0)	72.1 (68.2–81.3)	63.8 (58.2–86.4)

Data are presented as median (IQR) and percentage variation of the mean value with respect to control.

† *P* < 0.01 compared to control group

* *P* < 0.05 compared to control group

## Discussion

Joint immobilization at clinical sites is essential for injured patients, such as plaster casting or orthosis for traumatic diseases such as bone fracture or tendon rupture. A significant number of patients with osteoporosis also develop disuse osteopenia owing to being bedridden caused by aging-related cardiovascular and cerebrovascular diseases [1–3]. Osteocytes embedded in the bone matrix respond to mechanical stress on bones, such as from load-bearing and muscle traction force [25, 26]. Gap junctions in the long processes of osteocytes are thought to play an important role in transmitting mechanical stress [27] through intracellular signal transmitters [28] and extracellular signal transmitters [29] to induce bone formation by osteoblasts, inhibition of bone resorption by osteoclasts, or a combination of the two. The morphological characteristics of osteopenia are a decrease in bone mineral density and deterioration of cortical and cancellous bone structures, resulting in reduced bone strength and an increased risk of fragility fractures [30]. However, previous *in vivo* studies investigating osteopenia have confused the mechanical stressors of load-bearing and muscle traction force. Although unloading-induced osteopenia has been assessed using tail suspension models [31, 32], the mechanism of osteopenia caused solely by excluding joint motion remains largely unexplored. The present study examined temporal changes in both cortical and cancellous microstructure in immobilization osteopenia caused by load-bearing plaster cast fixation.

Because 8-week-old SPF Wistar rats used in this study were recognized to be in growth phase, length of the femur, and Tt.Ar of the midshaft increased during the experimental period over time. In the Im group, however, increment rates for these values were lower than those in the control group. These results indicate that fixation with a plaster cast inhibited normal growth in rats, including body weight and femoral elongation. After 1 week of immobilization, values of Tt.Ar and Ma.Ar in the diaphysis became lower than in the control group and this situation continued until 12 weeks. Similarly, Ct.Ar and Ct.Th became lower after 2 weeks. Loss of joint motion from muscle contracture presumably led to decreases in overall bone size and bone marrow at 1 week and thinning of the cortex followed after 2 weeks of immobilization. The value of pMOI in the Im group also decreased after 2 weeks compared to the control group and this trend continued until the end of the experiment. Generally, as osteoporosis progressed, cortical bone became thinner and more porous, and pMOI enhancing structural strength decreased [33]. Although cortical porosity was not evaluated in this study, the results for pMOI indicate that immobilization increases fragility of the femoral diaphysis in only 2 weeks. Yarrow et al. reported 3D morphometric measurements from a cast immobilization rodent model and significant differences could not be defined in cortical structural parameters after 2 weeks [34]. This inconsistency may be attributable to differences in animal age and region of analysis by micro-CT. They used 16-week-old rats and scanned the distal femoral metaphysis, in which some osteoporotic changes and structural deterioration could already have been present.

In terms of the cancellous microstructure of the distal femoral metaphysis, BV/TV and Tb.Th in the Im group decreased and SMI increased significantly after 2 weeks compared to the control group, and differences widened as the period of immobilization continued. These findings indicate that trabecular thickening was hindered, and trabecular structures changed from plate-like to rod-like characteristics by cast immobilization, resulted in loss of trabecular bone volume. Conn.D became lower in the Im group than in the control group at 12 weeks, and DA and Tb.Sp shifted in the same manner until the end of the experiment. Generally, aging and postmenopausal osteoporosis result in decreasing BV/TV, Conn.D, and Tb.N, and increasing SMI, Tb.Sp, and structural heterogeneity in cancellous bone [35, 36]. From a trabecular microstructural viewpoint, the changes in immobilization osteopenia are similar to those in osteoporosis, but the decreases to BV/TV and Tb.Th and increases to SMI in the early phase are characteristic. As for bone metabolism, trabecular bone is approximately eight times as active as cortical bone, because the surface area of trabecular bone is much larger than that of cortical bone, and its response to metabolic changes is thus faster [37]. Images from 3D micro-CT showed sparse structures of trabecular bone, particularly in the core of bone marrow. Trabeculae near the endocortical surface (subcortical spongiosa) are considered to play an important role in sustaining weight loading [36], so marked bone loss might be evident in the core region in the present rodent model which allowed weight-bearing. In a rat model of sciatic neurectomy, which excluded joint mobility, weight-bearing, and autonomic function, Ito et al. found marked reductions in BV/TV, Tb.Th, Tb.N, and DA and a rise in Tb.Sp after 4 weeks [35]. Ju et al. reported that the same trends in microstructural parameters were evident after 2 weeks in the femora of tail suspension rats [31]. A hind limb unloading mouse model displayed a 68.6% loss of trabecular BV/TV in 2 weeks [30] and an inactive rat model showed significant decreases in Ct.Ar, Ma.Ar, and BV/TV after 15 weeks [38]. From the perspective of bone microstructure, mechanical stress from muscle contracture is thought to assume a key role in osteopenia, and immobilization causes microstructural deterioration in both cortical and cancellous bone after 2 weeks, showing effects similar to those under unloading conditions. Skeletal muscle atrophy and muscle weakness caused by immobilization might also diminish mechanical stress on bone and exacerbate osteopenia. The biomechanical and biochemical interactions between muscle and bone are currently a matter of focus [10]. Muscle-derived forces are transmitted to the skeleton to produce mechanical loading at the end of the lever arm (bone) [39]. Besides that, muscle-secreted factors such as interleukin 15 and irisin increase bone mineral content and promote osteoblast differentiation [10, 40, 41]. It should be noted, therefore, that immobilization tolerating load-bearing, such as brace fixation for vertebral compression fracture or following spinal surgery, or fixation of lower extremities with a cylinder-type cast or knee brace, can induce rapid periosteal and endosteal bone resorption and evoke deterioration of bone microstructure as a result. In particular, the human upper extremities, for which mechanical stress mostly depends on muscle contracture, may be at amplified risk of fracture from immobilization. Yokota et al. reported that both trabecular and cortical bone microstructure showed marked deterioration with age [42]. Immobilization-induced osteopenia caused by sarcopenia in the elderly might be partially associated with age-related deterioration of bone microstructure.

Since the present study was not designed to elucidate the mechanisms of action of the combined treatment, the precise cellular mechanisms of action remain unclear. Patterns of bone deterioration vary in different regions [43], and the results of this study are thus not necessarily applicable to all bones in the body. Moreover, whole bone strength is determined by both bone mass and bone quality [43] and the architectural results of this study do not directly reflect the mechanical properties of bone. To elucidate the mechanisms underlying immobilization osteopenia in greater detail, quantitative assessment by multifaceted evaluation is essential, including food consumption and activity. Biomechanical strength tests and molecular biological approaches should be considered in future studies, along with bone metabolic markers, crystal-chemical characteristics of apatite, and pathological approaches including immunostaining.

## Conclusions

Sequential changes to the 3D bone microstructure of load-bearing immobilization osteopenia were evaluated in a fixed limb rat model. Femoral bone showed inhibition of normal growth and progression of microstructural deterioration in both cortical and cancellous bone. The architectural vulnerability of bone resulting from lack of muscle contracture arose after 2 weeks in both the metaphysis and midshaft.

## Supporting information

S1 Data (ZIP)

S2 Data Representative values of body weight of rats. (ZIP)

S1 File (PDF)

## Abbreviations

SPF: specific-pathogen-free
micro-CT: micro-computed tomography
3D: 3-dimensional
Tt.Ar: total area
Ma.Ar: marrow area
Ct.Ar: cortical bone area
Ct.Ar/Tt.Ar: cortical bone area fraction
Ct.Th: cortical thickness
pMOI: polar moment of inertia
BV/TV: bone volume fraction
Tb.Th: trabecular thickness
Tb.Sp: trabecular separation
Tb.N: trabecular number
SMI: structural model index
DA: degree of anisotropy
Conn.D: connective density
