# Supplementary material for: Progression of microstructural deterioration in load-bearing immobilization osteopenia
Source: PLoS One. 2022 Nov 4;17(11):e0275439. doi: 10.1371/journal.pone.0275439 (PMC9635731; doi:10.1371/journal.pone.0275439)
Supplement: S1 Data — (ZIP) [file pone.0275439.s001.zip › ÉVé╡éóâtâHâïâ_ü[ (2)/Diaphysis.pdf]

number	fixation	period	dTt.Ar	dMa.Ar	dCr.Ar	dCtAr TtAr(%)	dCtTh	dCtTh(μm)	dpMOI
1	1	1	10.06564	6.12676	5.94237	59.03618647	0.562	562	13.61941
2	1	1	10.03077	6.13082	5.93116	59.12965804	0.549	549	13.563
3	1	1		5.93732	5.85986		0.508	508	18.2442
4	1	1		5.98572	5.86365		0.501	501	18.25844
5	1	1	10.00358	5.10222	5.0217	50.19902875	0.505	505	12.28003
6	1	1	10.15944	5.30747	5.24002	51.57784287	0.531	531	12.75652
7	1	1	9.0843	5.21728	5.0861	55.98780313	0.52	520	10.68575
8	1	1	9.7103	5.31931	5.21056	53.66013408	0.511	511	11.89306
9	1	1	9.24917	5.24438	5.11285	55.27901423	0.501	501	10.9342
10	1	1	9.12862	5.09008	4.9213	53.91066777	0.477	477	10.49356
11	1	1	9.9342	5.13581	5.04109	50.74480079	0.483	483	12.06446
12	1	1	10.54835	5.40335	5.29181	50.16718255	0.472	472	13.45961
13	2	1	11.12471	6.45051	6.36933	57.25389696	0.625	625	16.88041
14	2	1	10.21873	6.33787	6.26633	61.32200381	0.652	652	14.73383
15	2	1		7.82734	7.65895	54.04752176	0.602	602	
16	2	1		8.02809		57.62029919	0.6	600	
17	2	1	12.69349	7.80569	7.71022	60.74152971	0.695	695	21.97921
18	2	1	12.33475	7.95838		63.53578305			21.04408
19	2	1	10.5614	6.3203	6.18325	58.54574204	0.53	530	14.60603
20	2	1	10.73032	6.37436	6.21425	57.91299793	0.533	533	15.08809
21	2	1	8.71737	4.9774	4.89865	56.19412736	0.528	528	9.91307
22	2	1	9.24911	4.90844	4.8352	52.27746237	0.489	489	10.78151
23	2	1	10.41949	5.40743	5.29537	50.82177727	0.497	497	13.36962
24	2	1	10.25954	5.52183	5.41238	52.75460693	0.495	495	13.25451
25	1	2	9.33856	5.09024	4.98873	53.42076294	0.509	509	11.01709

26	1	2	9.55267	5.11604	5.02834	52.63805826	0.526	526	11.49387
27	1	2	9.64328	5.12292	4.93473	51.17273376	0.432	432	11.33342
28	1	2	9.73204	5.35231	5.1781	53.20672747	0.463	463	11.8452
29	1	2	8.611392	4.611392	5.532031	59.5958419	0.5711	571.06429	9.989013
30	1	2	8.686545	4.686545	4.970228	52.61272097	0.5257	525.65042	9.458227
31	1	2	11.33909	7.339091	5.621459	46.04830469	0.4753	475.26887	14.50374
32	1	2	10.29513	6.295131	5.986987	54.26824901	0.5773	577.34183	13.55452
33	1	2	9.770619	5.770619	5.371657	50.88374898	0.5093	509.33172	11.63778
34	1	2	8.356668	4.356668	5.40574	59.90115466	0.4795	479.49546	
35	1	2	8.394655	4.394655	4.842509	52.92068898	0.5354	535.39762	
36	1	2	10.15752	6.157517	5.729011	52.4637112	0.5841	584.10592	12.82537
37	1	2	9.874202	5.874202	5.567672	52.33508797	0.5562	556.22638	12.21752
38	1	2	10.11957	6.119568	5.556541	50.95613707	0.5254	525.44048	12.57998
39	2	2	10.077	5.78193	5.66162	56.18358638	0.536	536	13.26578
40	2	2	10.56295	5.75976	5.9628	53.28208502	0.514	514	14.01911
41	2	2	10.1815	6.24578		58.56504444	0.505	505	13.9858
42	2	2	10.35069	5.86762	7.700321	54.65712914	0.483	483	13.78509
43	2	2	15.27481	5.410295	6.425485	47.79321277	0.6314	631.43502	27.66372
44	2	2	9.410295	6.993509	6.236243	64.03077049	0.6191	619.14323	12.44877
45	2	2	10.99351	6.949393	6.140859	53.08807976	0.5988	598.7913	15.29663
46	2	2	10.94939	6.670168	6.172581	52.4308469	0.5758	575.75957	15.13923
47	2	2	10.67017	5.810328	5.799167	54.10019053	0.5806	580.61753	14.64775
48	2	2	9.810328	7.94959	6.837457	55.035533	0.6531	653.05505	12.3879
49	2	2	11.94959			53.87178044			17.80387
50	2	2	10.98092	6.980918	6.275621	53.50755491	0.6319	631.87516	15.22666
51	2	2	10.69283	6.692833	6.001506	52.38561084	0.5908	590.75569	14.43945

52	2	2	10.77524	6.775242	6.313364	54.8791778	0.6217	621.67602	14.9777
53	1	4	10.11797	6.41432	6.34186	62.67917379	0.646	646	14.6395
54	1	4	10.43707	6.28337	6.15434	58.96616579	0.601	601	14.72677
55	1	4	9.76998	5.64518	5.53208	56.62324795	0.525	525	12.49603
56	1	4	9.61572	5.45961	5.3602	55.74413564	0.525	525	11.91083
57	1	4	9.43542	5.82109	5.68517	60.25349163	0.566	566	12.45485
58	1	4	10.1593	5.73803	5.61367	55.25646452	0.563	563	13.53628
59	1	4	9.49455	5.38236	5.27403	55.54797226	0.567	567	11.65813
60	1	4	9.2777	5.24918	5.13264	55.32233204	0.547	547	10.99251
61	1	4	9.3666	5.67384	5.56495	59.41270045	0.581	581	11.90578
62	1	4	9.0904	5.54954	5.43021	59.7356552	0.563	563	11.19065
63	1	4	10.88261	6.2089	6.0662	55.74214274	0.566	566	15.3646
64	1	4	11.23117	6.06697	5.93946	52.88371559	0.547	547	16.03764
65	2	4	15.06468	8.48943	8.37069	55.5650037	0.664	664	29.16823
66	2	4	14.6394	8.67558	8.50972	58.12888506	0.662	662	28.42277
67	2	4	12.98126	7.47804	7.34435	56.57655728	0.622	622	21.80609
68	2	4	13.12204	7.37376	7.28228	55.49655389	0.639	639	21.90437
69	2	4	11.85961	6.76366	6.66371	56.18827263	0.634	634	18.31254
70	2	4	11.64377	6.73695	6.62178	56.86972518	0.634	634	17.72244
71	2	4	11.66911	6.46869	6.37177	54.60373585	0.608	608	17.47975
72	2	4	11.93176	6.94974	6.84362	57.35633301	0.647	647	18.79604
73	2	4	11.82833	6.75752	6.59161	55.72730893	0.587	587	18.15602
74	2	4	12.26555	6.72116	6.61014	53.89191679	0.605	605	19.1199
75	1	8	9.19527	5.99042	5.88293	63.97778423	0.669	669	11.82811
76	1	8	9.37456	5.86483	5.76338	61.47893874	0.64	640	12.13125
77	1	8	9.6605	6.22736	6.13315	63.48687956	0.664	664	13.5172

78	1	8	9.92146	6.37543	6.23256	62.81898027	0.619	619	13.8822
79	1	8	11.65483	6.85922	6.75354	57.94627635	0.638	638	17.92984
80	1	8	11.5727	6.60836	6.50132	56.17807426	0.636	636	17.28412
81	1	8	12.28049	7.27555	7.17554	58.43040465	0.666	666	20.09318
82	1	8	12.28298	7.29268	7.20261	58.63894592	0.69	690	20.05821
83	1	8	12.79023	7.30172	7.18163	56.14934212	0.656	656	21.56858
84	1	8	13.01882	7.13116	7.03012	53.99967125	0.626	626	21.93124
85	1	8	12.57173	7.43723	7.31655	58.19843411	0.669	669	20.69457
86	1	8	12.82867	7.1088	7.00948	54.63917928	0.636	636	21.03577
87	2	8	11.85199	7.64041	7.61182	64.22398264	0.742	742	19.33728
88	2	8	11.938	7.54189	7.51628	62.96096499	0.732	732	19.44672
89	2	8	12.87165	8.3741	8.40629	65.30856572	0.768	768	23.03758
90	2	8	13.32234	8.05732	8.12349	60.97645008	0.731	731	24.04772
91	2	8	12.37757	7.43374	7.29316	58.92238945	0.673	673	20.77075
92	2	8	13.44115	7.77651	7.68109	57.14607753	0.686	686	24.14469
93	2	8	14.11059	8.03548	7.91881	56.11962363	0.692	692	26.01476
94	2	8	14.47669	7.90721	7.78677	53.78833145	0.67	670	26.47523
95	2	8	14.99763	8.82342	8.70789	58.06177376	0.732	732	30.0344
96	2	8	14.98681	8.97867	8.84204	58.99881296	0.749	749	30.30823
97	2	8	15.59569	9.01538	8.88375	56.9628532	0.721	721	31.56117
98	2	8	15.82575	9.06674	8.94646	56.53103328	0.733	733	32.42177
99	2	8	15.41946	8.57814	8.43969	54.73401792	0.689	689	31.37419
100	2	8	14.61307	8.3395	8.22521	56.28666666	0.694	694	28.34643
101	1	12	10.772	6.97669	6.86476	63.72781285	0.725	725	16.12853
102	1	12	11.81772	6.83214	6.71178	56.79420396	0.637	637	18.35485
103	1	12	14.03282	7.62355	7.498	53.43188326	0.656	656	24.63052

104	1	12	11.78273	6.32894	6.21586	52.75398825	0.589	589	17.49884
105	1	12	11.46447	6.37924	6.25468	54.55707939	0.606	606	16.77459
106	2	12	14.79433	8.39958	8.2891	64.7872925	0.781	781	23.18869
107	2	12	14.28272	8.87792	8.75148	61.27320286	0.784	784	27.75061
108	2	12	13.46941	8.53647	8.40389	62.39241362	0.774	774	25.44433
109	2	12	13.69271	8.73602	8.61259	62.8990901	0.781	781	26.38778
109	2	12	14.03282						
