# Supplementary material for: Progression of microstructural deterioration in load-bearing immobilization osteopenia
Source: PLoS One. 2022 Nov 4;17(11):e0275439. doi: 10.1371/journal.pone.0275439 (PMC9635731; doi:10.1371/journal.pone.0275439)
Supplement: S1 Data — (ZIP) [file pone.0275439.s001.zip › ÉVé╡éóâtâHâïâ_ü[ (2)/Metaphysis.pdf]

number	fixation	period	mBVTV	mBVTV(%)	mConnDens	mSMI	mTbN	mTbTh	mTbTh(μm)	mTbSp	mTbSp(μm)	mDA
1	1	1	0.2533	25.33	66.5882	1.6132	3.7693	0.0953	95.3	0.256	256	1.6839
2	1	1	0.2457	24.57	67.4475	1.7101	3.735	0.0926	92.6	0.2513	251.3	1.6964
3	1	1	0.2174	21.74	62.3633	1.7684	3.5295	0.0864	86.4	0.2713	271.3	1.7328
4	1	1	0.2314	23.14	70.0785	1.6595	3.5692	0.0881	88.1	0.2694	269.4	1.7123
5	1	1	0.3029	30.29	81.2634	1.0259	4.098	0.0914	91.4	0.231	231	1.6867
6	1	1	0.354	35.4	91.1499	0.6934	4.424	0.0975	97.5	0.2028	202.8	1.734
7	1	1	0.3973	39.73	107.8273	0.197	4.3815	0.0922	92.2	0.2225	222.5	2.127
8	1	1	0.3833	38.33	97.2041	0.4765	4.6672	0.094	94	0.2007	200.7	2.2341
9	1	1	0.3155	31.55	86.7445	1.1401	4.9796	0.0942	94.2	0.1823	182.3	2.1107
10	1	1	0.3198	31.98	93.2421	1.0144	4.9157	0.0963	96.3	0.1932	193.2	1.9952
11	1	1	0.3513	35.13	104.1333	0.8917	4.0538	0.0954	95.4	0.2364	236.4	2.0578
12	1	1	0.389	38.9	104.0188	0.5298	4.2496	0.0945	94.5	0.2198	219.8	2.1464
13	2	1	0.3556	35.56	81.4463	0.4895	4.0832	0.1005	100.5	0.2311	231.1	1.9992
14	2	1	0.34	34	68.4115	0.47	3.7754	0.1028	102.8	0.2655	265.5	2.0475
15	2	1	0.36	36	103.9826	0.2248	4.3289	0.0987	98.7	0.2215	221.5	1.9418
16	2	1	0.2629	26.29	71.5341	1.3293	3.6385	0.0943	94.3	0.2631	263.1	1.8999
17	2	1	0.2891	28.91	70.2141	1.2578	3.69	0.0985	98.5	0.2598	259.8	2.0084
18	2	1	0.3558	35.58	77.1904	0.7229	4.2604	0.1044	104.4	0.2155	215.5	1.9468
19	2	1	0.3371	33.71	84.9866	0.6729	5.4075	0.0943	94.3	0.1641	164.1	2.003
20	2	1	0.3613	36.13	93.3139	0.5659	5.2991	0.0944	94.4	0.1674	167.4	1.9686
21	2	1	0.3735	37.35	95.959	0.4736	4.7086	0.0913	91.3	0.1939	193.9	1.9544
22	2	1	0.3891	38.91	90.8562	0.1139	4.713	0.0896	89.6	0.1924	192.4	1.9705
23	2	1	0.3136	31.36	76.3381	0.9874	5.063	0.0891	89.1	0.1695	169.5	2.1347
24	2	1	0.3209	32.09	80.4022	1.024	5.2268	0.0948	94.8	0.1622	162.2	2.1425
25	1	2	0.1926	19.26	54.4138		3.3965	0.0777	77.7	0.2484	248.4	1.9276

26	1	2	0.2248	22.48	63.2817	1.7252	3.8123	0.0801	80.1	0.2637	263.7	1.9563
27	1	2	0.2572	25.72	72.2261	1.4339	3.7127	0.0875	87.5	0.249	249	1.7909
28	1	2	0.2565	25.65	74.7684	1.396	3.8769	0.0846	84.6			1.8457
29	1	2	0.157519	15.75185	48			0.09005	90.05127			2.25859
30	1	2	0.314924	31.49242	106			0.10241	102.41189	0.16994	169.94204	
31	1	2	0.32202	32.20202	119			0.10273	102.72881			1.72185
32	1	2	0.348048	34.80484			3.31	0.10518	105.18158			
33	1	2	0.328585	32.85848		1.68588	3.38	0.09736	97.3577	0.1822	182.19729	1.73363
34	1	2	0.279821	27.98205	101			0.10016	100.16155	0.19428	194.28238	
35	1	2	0.315768	31.57676	108			0.10194	101.93698	0.17838	178.37839	1.73299
36	1	2	0.358708	35.87077		1.73583	3.44	0.10415	104.15271			
37	1	2	0.358247	35.82469		1.65501	3.45	0.10373	103.72624	0.17153	171.53185	
38	1	2	0.320083	32.0083	119	1.74337		0.09733	97.33179	0.18124	181.23958	1.71984
39	2	2	0.4029	40.29	94.3252	0.3034	5.1174	0.0985	98.5	0.1724	172.4	2.1734
40	2	2	0.4298	42.98	92.585	-0.0151	5.1429	0.1055	105.5	0.1751	175.1	2.1497
41	2	2	0.3752	37.52	94.1627	0.4039	4.9365	0.0956	95.6	0.1824	182.4	
42	2	2	0.3121	31.21	77.7111	1.0592	4.3698	0.0932	93.2	0.2145	214.5	2.108
43	2	2	0.376	37.57652	90					0.18289	182.88998	1.76835
44	2	2	0.395	39.47783								
45	2	2	0.420	42.00496			3.65	0.11498	114.97984			
46	2	2	0.418	41.81894			3.86	0.10832	108.31936			
47	2	2	0.386	38.5757			3.31					1.64238
48	2	2	0.458	45.80399		0.73151	3.97					1.65147
49	2	2	0.549	54.94402		0.49225	3.55			0.13801	138.01205	
50	2	2	0.373	37.287								
51	2	2	0.415	41.46225			3.21	0.10731	107.3062	0.17468	174.68437	1.76576

52	2	2	0.324	32.44124	100	1.41058	3.86	0.11343	113.43329	0.1827	182.70415	
53	1	4	0.3438	34.38	73.9026	0.7664	4.4267	0.0992	99.2	0.2037	203.7	2.0564
54	1	4	0.3676	36.76	77.2804	0.4317	4.5104	0.1025	102.5	0.2065	206.5	1.9658
55	1	4	0.2703	27.03	79.2841	1.5101	4.2832	0.0854	85.4	0.2144	214.4	1.9555
56	1	4	0.2615	26.15	80.1038	1.5366	4.2287	0.0831	83.1	0.2202	220.2	2.0344
57	1	4	0.2193	21.93	59.0871	1.912	3.5967	0.0888	88.8	0.2595	259.5	1.7716
58	1	4	0.25	25	69.6279	1.6248	3.9402	0.0878	87.8	0.2366	236.6	1.7537
59	1	4	0.3665	36.65	95.3704	0.6446	4.88	0.0967	96.7	0.1838	183.8	1.7244
60	1	4	0.3365	33.65	90.2938	0.9432	4.8045	0.0922	92.2	0.1858	185.8	1.7729
61	1	4	0.2284	22.84	58.511	1.6637	3.2795	0.0902	90.2	0.3028	302.8	2.0244
62	1	4	0.2588	25.88	71.0538	1.4805	3.5743	0.0914	91.4	0.2746	274.6	1.9626
63	2	4	0.4339	43.39	0.3438	-0.0851	5.3138	0.1044	104.4	0.1585	158.5	1.7593
64	2	4	0.4573	45.73	0.3676	-0.2586	5.5119	0.1073	107.3	0.1498	149.8	1.8071
65	2	4	0.362	36.2	0.2703	0.56	4.4075	0.1019	101.9	0.2138	213.8	2.0164
66	2	4	0.351	35.1	0.2615	0.553	4.2158	0.1011	101.1	0.2265	226.5	2.0466
67	2	4	0.3949	39.49	0.2193	0.1232	4.3409	0.1094	109.4	0.226	226	1.9511
68	2	4	0.3572	35.72	0.25	0.6131	4.6925	0.0953	95.3	0.1918	191.8	2.1144
69	2	4	0.3245	32.45	0.3665	1.1131	4.2784	0.1008	100.8	0.2102	210.2	1.839
70	2	4	0.282	28.2	0.3365	1.4862	4.0168	0.0963	96.3	0.2266	226.6	1.8254
71	2	4	0.3072	30.72	0.2284	1.2528	3.9398	0.1043	104.3	0.2259	225.9	1.7656
72	2	4	0.2837	28.37	0.2588	1.4648	3.9727	0.0964	96.4	0.2215	221.5	1.801
73	1	8	0.3433	34.33	82.388	0.7062	3.9584	0.104	104	0.2497	249.7	1.7337
74	1	8	0.2932	29.32	82.7451	1.1099	3.7417	0.0941	94.1	0.2666	266.6	1.6719
75	1	8	0.2451	24.51	69.6289	1.7096	3.7693	0.0885	88.5	0.2505	250.5	1.7081
76	1	8	0.2803	28.03	74.4388	1.4296	3.9713	0.0941	94.1	0.2354	235.4	1.7415
77	1	8	0.2854	28.54	72.1378	1.2388	3.8704	0.0948	94.8	0.2414	241.4	1.6339

78	1	8	0.2397	23.97	65.5453	1.6494	3.7241	0.0889	88.9	0.2534	253.4	1.6639
79	1	8	0.3453	34.53	69.1507	0.9245	3.7798	0.1112	111.2	0.2284	228.4	1.7723
80	1	8	0.3439	34.39	62.5056	0.7504	3.6413	0.1132	113.2	0.249	249	1.7816
81	1	8	0.3616	36.16	86.4336	0.5834	4.1662	0.1071	107.1	0.2226	222.6	1.6465
82	1	8	0.3221	32.21	80.9451	0.9617	3.9998	0.101	101	0.232	232	1.654
83	1	8	0.2194	21.94	41.2337	1.9943	2.9118	0.1075	107.5	0.3371	337.1	1.5523
84	1	8	0.3076	30.76	71.9961	1.2986	3.8724	0.1045	104.5	0.2352	235.2	1.7099
85	2	8	0.3385	33.85	71.8951	0.2858	3.3795	0.1066	106.6	0.3086	308.6	1.6664
86	2	8	0.3335	33.35	71.7221	0.497	3.6179	0.108	108	0.283	283	1.6193
87	2	8	0.4289	42.89	81.3898	-0.112	4.4578	0.1181	118.1	0.2035	203.5	1.7691
88	2	8	0.3852	38.52	74.8199	0.1834	4.2755	0.1106	110.6	0.2237	223.7	1.7013
89	2	8	0.3138	31.38	60.6338	0.7381	3.3075	0.1126	112.6	0.3102	310.2	1.7485
90	2	8	0.3821	38.21	69.6942	0.27	3.9447	0.1174	117.4	0.2476	247.6	
91	2	8	0.3633	36.33	75.2263	0.3803	3.9744	0.1076	107.6	0.2478	247.8	1.7964
92	2	8	0.2913	29.13	63.0507	1.0151	3.2844	0.1044	104.4	0.3008	300.8	1.8409
93	2	8	0.2384	23.84	50.444	1.483	2.8689	0.1036	103.6	0.3601	360.1	1.791
94	2	8	0.3024	30.24	65.3648	1.0597	3.3622	0.1088	108.8	0.3067	306.7	1.7792
95	2	8	0.423	42.3	78.6572	0.033	4.4346	0.1174	117.4	0.2008	200.8	1.8023
96	2	8	0.4844	48.44	74.7269	-0.8999	4.7424	0.1299	129.9	0.1912	191.2	1.7922
97	2	8	0.4049	40.49	77.906	0.1521	4.2613	0.1156	115.6	0.2207	220.7	1.7735
98	2	8	0.423	42.3	79.1515	-0.1533	4.1857	0.1207	120.7	0.2312	231.2	1.8212
99	1	12	0.2176	21.76	57.1484	1.6869	2.8947	0.0922	92.2	0.3392	339.2	1.6985
100	1	12	0.1883	18.83	44.5305	1.8102	2.3117	0.0909	90.9	0.4494	449.4	1.7803
101	1	12	0.39	39	94.6946	0.3315	4.6526	0.103	103	0.1856	185.6	1.6508
102	1	12	0.3852	38.52	99.0406	0.4002	4.8283	0.1	100	0.1813	181.3	1.5881
103	1	12	0.3084	30.84	66.0386	1.015	3.6372	0.1058	105.8	0.2657	265.7	1.6799

104	1	12	0.2433	24.33	50.4013	1.4633	2.8897	0.1023	102.3	0.3531	353.1	1.6273
105	1	12	0.2614	26.14	61.1847	1.6572	3.2986	0.108	108	0.2825	282.5	1.6675
106	1	12	0.2617	26.17	61.5479	1.59	3.3219	0.1033	103.3	0.2867	286.7	1.7757
107	1	12	0.318	31.8	79.6425	0.9152	3.886	0.0999	99.9	0.2421	242.1	1.6338
108	1	12	0.3158	31.58	88.6374	0.8908	4.0404	0.0949	94.9	0.2376	237.6	1.6605
109	2	12	0.5148	51.48	89.9408	-1.231	5.39	0.1207	120.7	0.1632	163.2	1.7825
110	2	12	0.5588	55.88	90.3457	-1.8768	5.6393	0.1259	125.9	0.148	148	1.8281
111	2	12	0.5002	50.02	75.9707	-1.351	4.5986	0.134	134	0.2107	210.7	1.6505
112	2	12	0.5005	50.05	79.5894	-1.2521	4.816	0.1286	128.6	0.1945	194.5	1.7
113	2	12	0.4387	43.87	82.372	-0.4661	4.302	0.1175	117.5	0.2212	221.2	1.7544
114	2	12	0.3719	37.19	65.8515	0.2143	3.7712	0.1139	113.9	0.2697	269.7	1.7455
115	2	12	0.4028	40.28	48.7646	-0.2703	3.5096	0.1342	134.2	0.2824	282.4	1.6321
116	2	12	0.4048	40.48	56.506	-0.3233	3.3907	0.1327	132.7	0.3029	302.9	1.6433
117	2	12	0.4734	47.34	77.537	-0.9781	4.517	0.124	124	0.2154	215.4	1.6248
118	2	12	0.4849	48.49	75.3356	-1.272	4.455	0.1262	126.2	0.2218	221.8	1.626
