# Supplementary material for: Progression of microstructural deterioration in load-bearing immobilization osteopenia
Source: PLoS One. 2022 Nov 4;17(11):e0275439. doi: 10.1371/journal.pone.0275439 (PMC9635731; doi:10.1371/journal.pone.0275439)

groups	week	body weight	Ave	SD
con	1w	327	317.0	10.1
con	1w	320		
con	1w	320		
con	1w	300		
con	1w	318		
con	2w	350	366.0	15.2
con	2w	390		
con	2w	360		
con	2w	360		
con	2w	370		
con	4w	430	420.8	19.5
con	4w	410		
con	4w	392		
con	4w	432		
con	4w	440		
con	8w	455	504.0	34.5
con	8w	480		
con	8w	525		
con	8w	530		
con	8w	530		
con	12w	626	609.2	37.1
con	12w	543		
con	12w	630		
con	12w	625		
con	12w	622		
Im	1w	248	257.0	5.7
Im	1w	260		
Im	1w	260		
Im	1w	255		
Im	1w	262		
Im	2w	280	276.2	28.0
Im	2w	315		
Im	2w	250		
Im	2w	288		
Im	2w	248		
Im	4w	288	297.2	36.7
Im	4w	245		
Im	4w	341		
Im	4w	290		
Im	4w	322		
Im	8w	312	342.4	23.2
Im	8w	375		
Im	8w	352		
Im	8w	338		
Im	8w	335		
Im	12w	395	357.5	32.3
Im	12w	320		
Im	12w	345		
Im	12w	370		
Im	12w	345		

	1W	2W	4W	8W	12W
con	317	366	420.8	504	609.2
Im	257	276.2	297.2	342.4	357.5
con	10.0995	15.16575	19.52434	34.53259	37.11738
Im	5.656854	28.02142	36.72465	23.20129	32.27486

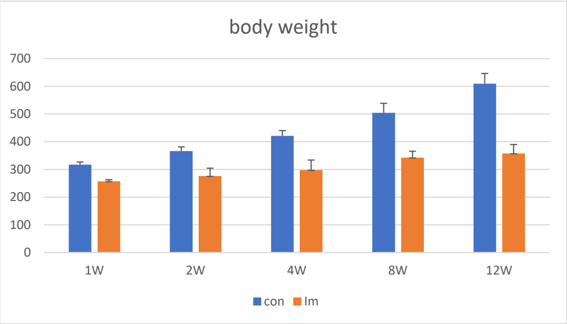

Supplement: S2 Data — (ZIP) [file pone.0275439.s002.zip › ÉVé╡éóâtâHâïâ_ü[ (2)/Body weight data.pdf]
