# Supplementary material for: Progression of microstructural deterioration in load-bearing immobilization osteopenia
Source: PLoS One. 2022 Nov 4;17(11):e0275439. doi: 10.1371/journal.pone.0275439 (PMC9635731; doi:10.1371/journal.pone.0275439)
Supplement: S1 File — (PDF) [file pone.0275439.s003.pdf]

メッセージを日本語に翻訳する | 英語からは翻訳しない

plosone <plosone@plos.org>
宛先: 小関　弘展; 尾崎　誠; 本田　祐一郎; shinyaknot@yahoo.co.jp; g.f.jiroko3@gmail.com; kiyukata1219@yahoo.co.jp; umirajh327@outlook.jp; 坂本　淳哉; 朝永　育 他 3 名

2022/09/27 (火) 14:33

Dear authors,

We received a request to amend the author list on the PLOS ONE manuscript, "Progression of microstructural deterioration in load-bearing immobilization osteopenia" (PONE-D-22-17568). The corresponding author would like to update the byline as follows:

Hironobu Koseki*, Makoto Osaki, Yuichiro Honda, Shinya Sunagawa, Chieko Imai, Takayuki Shida, Umi Matsumura, Junya Sakamoto, Iku Tomonaga, Seiichi Yokoo, Satoshi Mizukami, Minoru Okita

Before I can move forward, I need approval from the following authors:

Satoshi Mizukami
Minoru Okita

1. The above authors: Please respond to this email and CC the corresponding author to confirm your agreement to the proposed author list.
2. The corresponding author: Please compile all of the positive responses from the above authors into a single PDF and return this file by email.

I can proceed once the file containing all positive responses has been received. I'm happy to help with any questions, and look forward to your response!

Best,
Tom Jeffrey C. Macalisang
Straive Editorial Assistant

PLOS ONE | plosone@plos.org
Empowering researchers to transform science

Case Number: 07736943
ref:_00DU0Ifis._5004P22rVIj:ref

メッセージを日本語に翻訳する | 英語からは翻訳しない

水上　諭
宛先: plosone <plosone@plos.org>
Cc: 小関　弘展

2022/09/27 (火) 14:59

Tom Jeffrey C. Macalisang
Straive Editorial Assistant
PLOS ONE

Thank you for your mail about ACTION REQUIRED for PONE-D-22-17568.

I agree to the proposed author list.

Yours sincerely,

Satoshi Mizukami

...

メッセージを日本語に翻訳する | 英語からは翻訳しない

沖田　実
宛先: plosone <plosone@plos.org>
Cc: 小関　弘展

2022/09/29 (木) 15:55

Dear Tom Jeffrey C. Macalisang
(CC. Corresponding author; Prof. Koseki)

Sorry for the late reply.
I approve as a co-author of the PLOS ONE manuscript, "Progression of microstructural deterioration in load-bearing immobilization osteopenia" (PONE-D-22-17568).

Best regards

Prof. Minoru Okita
Nagasaki University

...

返信　全員に返信　転送
